# The urban heat island under extreme heat conditions: a case study of Hannover, Germany

**DOI:** 10.1038/s41598-023-49058-5

**Published:** 2023-12-27

**Authors:** Nadja Kabisch, Finja Remahne, Clara Ilsemann, Lukas Fricke

**Affiliations:** Institute for Physical Geography and Landscape Ecology, Leibniz University Hannover, Schneiderberg 50, 30167 Hannover, Germany

**Keywords:** Climate sciences, Environmental sciences, Environmental social sciences

## Abstract

Global warming has resulted in higher frequencies of climate extremes, such as drought periods or heat waves. Heat waves are intensified in urban areas due to the urban heat island effect. Studies are inconclusive as to whether the urban heat island effect is intensified during heat waves. Using the city of Hannover, Germany, as a case study, we analysed the intensity of the urban heat island under unprecedented summer heat conditions in the years 2018, 2019 and 2020, which were among the hottest in Germany since weather records began. We compared the intensity of the urban heat island across these years with the non-heat year of 2017. Differences were analysed for various inner-city urban locations and an urban park, while accounting for their distinct land use and land cover characteristics. We identified the urban heat island effect across all years investigated in the study and also found a significant intensified urban heat island effect during the years of unprecedented heat, when night-time temperature minima are considered. The urban heat island was identified on a lower level, however, with maximum daily temperatures when compared to the non-heat year. The lowest intensity of the urban heat island was found for the urban park site, highlighting the need for more city-wide greening strategies, including tree-covered and open green spaces, to provide all residents with the cooling services of green spaces.

## Introduction

For the past several decades, many regions around the world have been impacted by extreme climate events as a result of climate change, including extreme precipitation, droughts and heatwaves^1^. The impacts of these extreme events and, particularly, their co-occurrence with one another, such as heatwaves occurring under drought conditions, have been shown to result in severe environmental, economic, social and health challenges, as seen, for instance, in China^2^ and Europe^3^. Health-related challenges associated with heat periods include increased mortality and morbidity. For example, the summer heatwave in Europe in 2003 resulted in more than 70,000 heat-related deaths^4^and the heatwave in 2022 in more than 60,000 estimated deaths, predominantly in Spain and Germany^5^. Excess summer deaths were shown for Germany for the previous extreme heat years of 2018, 2019 and 2020, with approximately 20,000 heat-related deaths for this period^6^. In particular, the year 2018 was highlighted as the warmest year in Germany since the beginning of systematic weather records, with an annual average temperature of 10.5 °C being 2.3 °C higher than the long-term mean (1961–1990: 8.2 °C). Also, the years of 2019 (10.3 °C) and 2020 (9.9 °C) were exceptionally warm^7^. In fact, exceptionally high temperatures during these periods were identified for cities like Berlin and Hannover.

Cities are further impacted by the urban heat island (UHI) effect. The UHI is expressed by elevated temperatures within cities compared to their rural surroundings^8^. Higher temperatures in urban areas are caused by the specific characteristics of the urban landscape including a high degree of impervious surface, an accumulation of concrete material, the three-dimensionality of urban structures, limited shares of green and blue spaces, and anthropogenic heat storage and release, such as from cars, industry and air conditioning. The UHI is, thus, a result of the spatial arrangement of the built environment, the urban land use and land cover (LULC), overall thermal characteristics, and additional heat released from anthropogenic sources, all aggravated by ongoing urbanisation processes^9^.

As extreme climate events such as heat waves will occur in higher frequencies globally^1^, the possibility that the UHI effect may be intensified or amplified by heat waves (such as the above introduced heat waves occurring in Germany 2018, 2019 and 2020), through synergistic or combinative effects, should be examined. Scientific studies that analysed potential synergistic effects of UHI and heat wave conditions have used observational air temperature data from weather stations^10–12^ or land surface temperatures (LST) derived from remote sensing^13^ (for an overview of these different methodological approaches deriving UHI related effects see Lai et al.^14^ and Zhang et al.^15^). These studies mostly confirm the overall assumption of an intensified UHI during heat wave periods, e.g. for the cases of Madison, in the USA^16^, Melbourne and Adelaide, in Australia^10^, and Athens, in Greece^17^. For the Madison case, the authors found an intensification of the UHI for day-time and night-time during heat periods and that the UHI during day-time was stronger, with 39 days experiencing temperatures over 32.2 °C compared to only 9 days for the long-term mean^16^. In Melbourne, the UHI was identified to be more pronounced by up to 1.4 °C during heatwaves and in Adelaide by up to 1.2°C^10^.

The extent of an UHI intensification under heat was, however, shown to be very different depending on the respective climate zones^18^, local environmental conditions and LULC structures, and temporal aspects. For example, coastal cities, such as Athens, showed an amplification effect of the UHI under heat conditions with an average UHI by up to 3.5 °C, depending on the background wind field, such as sea breeze conditions which induce a cooling effect at coastal stations, intensifying the extent of the UHI^17^. In terms of LULC, urban green spaces are usually promoted as a nature-based solution to mitigate high temperatures^11,19–21^. Less is known, however, about the heat mitigation effect of urban green spaces during heat wave conditions. Finally, differences in the intensity of the UHI were reported with different temporal aspects, such as when focussing on different time periods throughout the day^17,22^. For example, synergistic effects of the UHI and a heat wave period were found particularly during the night but with a lower or even reversed effect during the day^10,23,24^. Some studies could not, however, identify any significant difference of the UHI intensity during heat wave periods compared to non-heat wave periods^25^ and most of the studies only assess one single heat event^16,17^.

In conclusion, the relationship between the UHI and heat, and their synergistic direction, is not fully conclusive and tends to depend on local, methodological, and temporal factors. The main aim of this study, then, is to assess the intensity of the UHI during several consecutive years of unprecedented extreme heat and to compare different urban built environments and an urban park location with the air temperature in rural areas. In particular, the following research questions are addressed:

What is the air temperature difference between certain urban areas and the rural surroundings in years of extreme summer heat conditions versus a non-extreme year? Are these potential differences significant?

To what extent does the potential intensity of the UHI differ when diversely-structured areas around urban measurement stations, including an urban park location, are considered?

Using the large German city of Hannover as a case, our objectives are to i) analyse air temperature data from five measurements stations in the rural, urban and urban park areas of the city for the summer time period of the years 2017–2020; ii) compare daily mean, maximum and minimum air temperature values for the summer period of the year 2017 as a non-heat year with the years of 2018, 2019 and 2020, known as years of extreme heat in Germany; iii) analyse the local LULC around the measurement stations to derive potential UHI intensity influencing factors.

## Methodology

### Case study: Hannover, Germany

The city of Hannover is the capital of the Federal State of Lower Saxony and is located close to the southern border of the Northern German Lowland in the transition area to the Lower Saxony Mountain and Hilly Region. The city is a large city counting 543,247 inhabitants in 2021, with a population density of 2,661 inhabitants/km^2^ and a total area of 204.15 km^2^^26^. The city has been growing, with a population growth rate of 7% between 2008 and 2018 and further growth is expected by 2.8% until 2029^27^.

**Figure 1:**
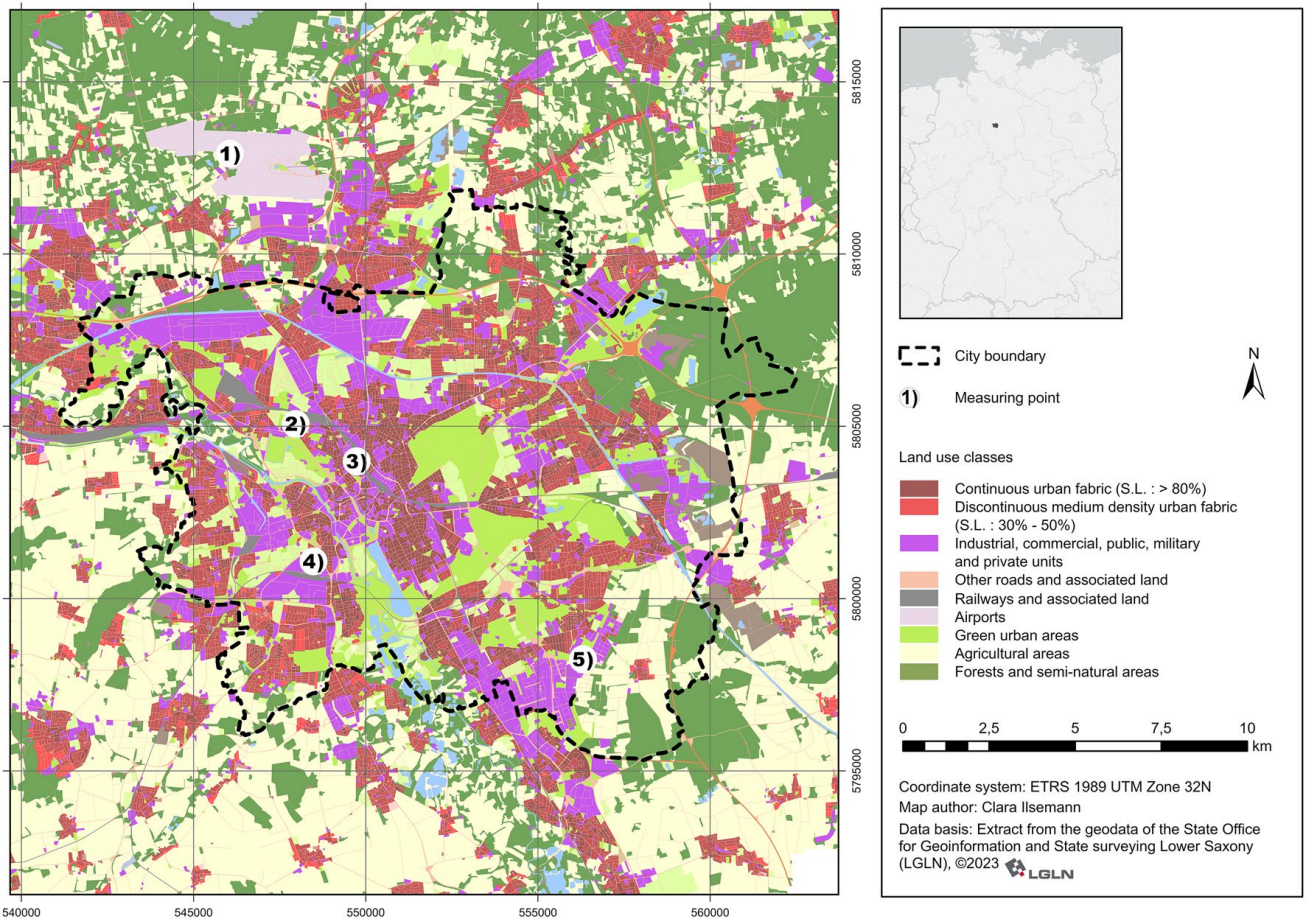
Hannover case study with land cover and location of the five measurement sites. Data source: Urban Atlas (EEA, 2018). Note: (1) LH = Langenhagen (rural site); (2) HE = Herrenhausen (urban site), (3) WD = Weidendamm (urban site); (4) MB = Marianne-Baecker Allee (urban site); (5) KP = Kattenbrookspark (urban park site).

Hannover is characterised by a diversity of different landcover categories within its 13 urban districts. A share of 35% of the city areas are dedicated as green spaces, including urban forests, parks, cemeteries, gardens, agricultural areas, etc. Blue spaces including lakes, canals, rivers, etc., accumulate to around 3.1% of the total city area (Fig. 1).

Hannover, like many other cities and regions in Germany, experienced unprecedented heat conditions during the summers of 2018, 2019 and 2020, when compared to previous years. Summer mean air temperature, yearly mean air temperature and sum of precipitation for the city of Hannover for the period 2010–2021 are presented in Table 1. The years of 2018–2020 stand out as the three hottest and also driest consecutive years. Similarly, the summer mean air temperatures in Germany for the years under study were: 2017: 17.9, 2018: 19.3, 2019: 19.2 and 2020: 18.2^28^. The year 2017, thus, is used as a comparison in our study, as a non-heat year when compared to the following consecutive years with unprecedented summer heat.

**Table 1:** Yearly mean air temperature, summer mean air temperature (June–August) and sum of precipitation for the city of Hannover for 2010–2021. Highlighted is the period 2017–2020 in bold. Source: DWD^29^.

Year	2010	2011	2012	2013	2014	2015	2016	2017	**2018**	**2019**	**2020**	2021
Yearly mean air temp. (°C)	8.5	10.4	9.9	9.4	11.1	10.6	10.3	10.4	**11.1**	**11.0**	**11.3**	9.9
Summer mean air temp. (°C)	18.6	17.2	17.2	18.0	17.5	18.1	18.3	17.8	**19.6**	**19.7**	**18.7**	18.4
Sum precip. (mm)	651	565	630	578	594	645	583	822	**434**	**582**	**494**	639

### Data

Data from a meteorological measurement campaign of the German Weather Service (GWS, DWD)^30^ are used in this study (Table 2). The meteorological measurement data were recorded with the help of temporary stationary measurements at various locations in urban and rural areas of the city of Hannover. Measured data included the following meteorological parameters: air temperature, relative humidity, wind speed, wind direction and, at selected locations, incoming global radiation, measured at a height of two meters over a period of three and a half years (June 2017–December 2020)^30^. As explained above, the focus of the years 2018–2020 is based on the fact that these years were shown to be the hottest years in Germany since weather records but also on data availability. The meteorological measurement campaign for the five stations ended in December 2020. Overall data loss was low (less than 1%), and data gaps or inconsistencies were reported only for one measurement station (HE urban) for some days in June 2018 (for the period 1.6–4.6; 7.6–10.6 and for the 12.6.–30.6.2018).

**Table 2:** Indicators and variables used for analysis.

Indicator	Variables	Data (source)
UHI	Daily mean air temperature Maximum air temperature Minimum air temperature	GWD 2022^30^
LULC	Percentage share of urban forest, green urban area within a 100 m buffer	Urban Atlas 2018^32^
	NDVI within a 100 m buffer	Digital Orthophoto, 2022, (DGO)^33^
	Number and percentage share of buildings of different heights within a 100 m buffer	3D building model, 2022^34^

For our analyses, we used the data for the summer period. The meteorological definition of the summer is from 1 June to 31 August. A summer day is defined as a day with a maximum air temperature ≥ 25 °C and a heat day is defined as a day with a maximum air temperature ≥ 30 °C. Finally, tropical nights are defined as nights where the minimum night temperature is ≥ 20 °C between 18:00 and 06:00 UTC^30^. We define the UHI intensity as the commonly used 2 m-height air temperature (near-surface air temperature) at each urban station minus the air temperature at the rural station, as per previous studies^23^. When using the maximum daily or minimum daily air temperatures, we also refer to them as day-time or night-time temperatures^31^.

The five measurement sites included three inner-city urban sites: Weidendamm (WD urban), Marianne-Baecker-Allee (MB urban), Herrenhausen (HE urban); an urban park site Kattenbrooks Park (KP urban park); and the rural site at Langenhagen (LH rural) close to the airport. The two stations HE and LH are under permanent use. The locations of the five stations are shown in Fig. 1.

The measurement station at Weidendamm (WD urban) is located in the Nordstadt district—a dense inner-city residential area. The Marianne-Baecker-Allee (MB urban) station is in the commercial area of the Linden-Süd district and can also be regarded as an inner-city location representing urban conditions. The Herrenhausen (HE urban) station is located on the premises of the Institute for Meteorology and Climatology of the Leibniz University Hannover. The measurement station in Kattenbrooks Park (KB urban park) is located within an open lawn area of an urban park. The measurement station representing the rural site is Langenhagen (LH rural), which is located northwest of the city. The location of the site complies with the internationally defined standards of the World Meteorological Organization (WMO)^30^.

To assess the LULC around the measurement stations, the Urban Atlas data of the European Environment Agency 2020 for Hannover was used with a 10 m minimum mapping width. A 3D-building model and a Digital Orthophoto were additionally used to analyse the local environmental and urban structures around the measurement stations (Table 1). The Digital Orthophoto with a grid width of 10 m presents the basis of the terrain heights of the buildings. The building model includes block models so that buildings are represented as rectangular and with flat roofs. The Digital Orthophoto (DOP), with a ground resolution of 20 cm covering  a total area of 2 km $\times$ 2 km, was used to calculate the Normalized Difference Vegetation Index (NDVI). The NDVI is an indicator for surrounding greenness^35^ and was previously used as an vegetation-indicating parameter in studies on the urban climate environment^36^.

### Data analysis

We applied descriptive statistics (mean, minimum and maximum, standard deviation) and spatial analyses in a Geographical Information System (GIS) to analyse the (differences in the) intensity of the UHI over the years and to show potential spatial–temporal differences and local specifics of the surrounding LULC.

A parametric test, the paired t-test, was used and considered appropriate for investigating the statistical significance in the outcome environmental variables between the rural site (LH rural), the different inner-city urban sites and the urban park site for each year of interest. A one-way analysis of variance (ANOVA) with post hoc tests (Bonferoni) for multiple comparisons were used to explore whether the UHI-intensity differs significantly between the years of interest, that is, to identify if there are significant differences in the UHI-intensity levels (measured by daily mean air temperature, max. air temperature and min. air temperature) of the non-extreme year (2017) compared to the extreme heat years (2018, 2019 and 2020). As a part of performing the ANOVA, tests for the required distributional assumptions were conducted which included the Levene statistics to assess the equal variance assumption. Significance for both the paired t-tests and the ANOVA was considered at the p < 0.05 level as applied in earlier studies^37^. Statistics were performed using IBM SPSS Statistics 28.0 (IBM Corporation, Armonk, NY, USA).

To assess the LULC in close vicinity of the measurement sites, a buffer of 100 m around the measurement stations was created to identify the percentage of the different LULC classes, the number and percentage of buildings of different heights, and percentage of the area within NDVI value classes. Buildings were grouped into three classes according to their height. The first class grouped buildings from 0 m to ≤ 13 m tall, including both detached buildings with no more than two units of use and detached buildings, such as those used for agriculture and forestry. The second class includes buildings such as single-family houses and high-rise buildings with heights between > 13 m and ≤ 22 m. The third class grouped high-rise buildings at a height > 22 m.

## Results

### Air temperature differences between the inner-city urban, the urban park and the rural measurement sites

Table 3 provides an overview of the environmental conditions during the summer periods for 2017–2020 in the city of Hannover. Mean air temperature was lowest in the non-heat year of 2017 for all measurement sites with lowest values of 17.8 °C for the rural site LH and the urban park site KP. Highest maximum air temperatures were measured for the heat year 2019, with the inner-city urban station HE, for instance, recording a maximum temperature of 39.6 °C in 2019. Mean values for relative humidity were usually highest for the rural site LH (with one exception of 2020 where the urban park (KP) area showed slightly higher values). 2017 stands out as the year with highest humidity values measured at all stations compared to the other years. Nearly all values measured at the urban and urban park stations were significantly different compared to the values at the rural station (LH rural).

**Table 3:** Descriptive statistics of air temperature, relative humidity and wind speed of the rural, urban and urban park measurement sites for the summer periods of 2017, 2018, 2019, 2020. Note: LH = Langenhagen, WD = Weidendamm, MB = Marianne-Baecker Allee, HE = Herrenhausen, KP = Kattenbooks Park.

	Year		LH (rural)	WD (urban)	MB (urban)	HE (urban)	KP (urban park)
Air temp. °C	2017	Mean (Std. dev.)	17.8 (2.2)	**18.7 (2.3)**	**18.6 (2.3)**	**18.6 (2.4)**	17.8 (2.2)
		Max./Min.	33.1/5.7	33.9/9.9	34.6/8.9	35.2/7. 3	33.3/5. 9
	2018	Mean (Std. dev.)	19.6 (3.6)	**20.7 (3.9)**	**20.7 (3.8)**	**20.7 (3.9)**	**19.9 (3.7)**
		Max./Min.	36.5/6.7	37.1/9.7	37.8/8.5	38.0/8.6	36.7/6.5
	2019	Mean (Std. dev.)	19.7 (3.4)	**20.9 (3.6)**	**20.7 (3.5)**	**20.7 (3.6)**	**20.0 (3.4)**
		Max./Min.	37.9/6.1	38.2/10.2	38.9/9.6	39.6/8.9	37.9/7.1
	2020	Mean (Std. dev.)	18.7 (3.5)	**19.8 (3.5)**	**19.7 (3.4)**	**19.6 (3.5)**	**18.9 (3.3)**
		Max./Min.	35.7/6.7	36.3/10.2	36.6/8.2	37.1/7.3	35.5 (7.2)
Rel. humidity %	2017	Mean (Std. dev.)	75.4 (9.2)	**67.5 (10.7)**	**67.8 (10.4)**	**72.7 (9.1)**	**73.2 (9.0)**
		Min./Max.	50.5 / 96.5	39.1/93.7	40.9/93.5	47.0/94.7	52.2/95.0
	2018	Mean (Std. dev.)	65.4 (11.5)	**57.4 (12.9)**	**59.9 (12.6)**	**62.5 (11.1)**	**62.8 (12.2)**
		Min./Max.	40.0/94.9	31.7/90.1	35.9/90.8	41.3/91.4	37.4/93.0
	2019	Mean (Std. dev.)	65.2 (10.4)	**58.2 (11.4)**	**61.9 (11.1)**	**64.2 (10.2)**	64.6 (11.0)
		Min./Max.	39.7/90.6	31.7/86.4	36.2/89.6	38.8/88.9	36.6/91.4
	2020	Mean (Std. dev.)	68.4 (11.3)	**64.3 (12.3)**	**64.2 (12.0)**	**67.4 (10.6)**	**69.9 (12.1)**
		Min./Max.	43.3/92.6	38.1/90.4	38.9/89.5	44.0/89.2	45.9/96.6
Wind speed* m/s	2017	Mean (Std. dev.)	3.4 (1.1)	**1.4 (0.4)**	**1.1 (0.3)**	**1.9 (0.7)**	**1.4 (0.5)**
		Min./Max	1.6/ 6.2	0.6/2.6	0.5/2.1	0.9/3.8	0.6/3.2
	2018	Mean (Std. dev.)	3.4 (1.1)	**1.4 (0.4)**	**1.1 (0.3)**	**1.8 (0.6)**	**1.4 (0.4)**
		Min./Max.	1.7/7.1	0.7/2.7	0.5/1.9	0.9/4.1	0.6/2.8
	2019	Mean (Std. dev.)	3.5 (1.1)	**1.4 (0.4)**	**1.0 (0.3)**	**1.9 (0.6)**	**1.4 (0.4)**
		Min/Max.	1.5/6.5	0.6/2.7	0.5/2.2	0.9/4.0	0.6/2.7
	2020	Mean (Std. dev.)	3.3 (1.2)	**1.3 (0.5)**	**1.0 (0.3)**	**1.8 (0.7)**	**1.3 (0.6)**
		Min./Max.	1.5/8.5	0.6/3.1	0.4/2.6	0.8/5.5	0.6/4.0

The mean values are shown in bold when they are significantly different from LH (rural) station at the 95% confidence level (t-test for difference between sample means).

*Wind speed was measured at 10 m height for LH (rural) and HE (urban) and at 2 m height for WD (urban), MB (urban) and KP (urban park).

**Figure 2:**
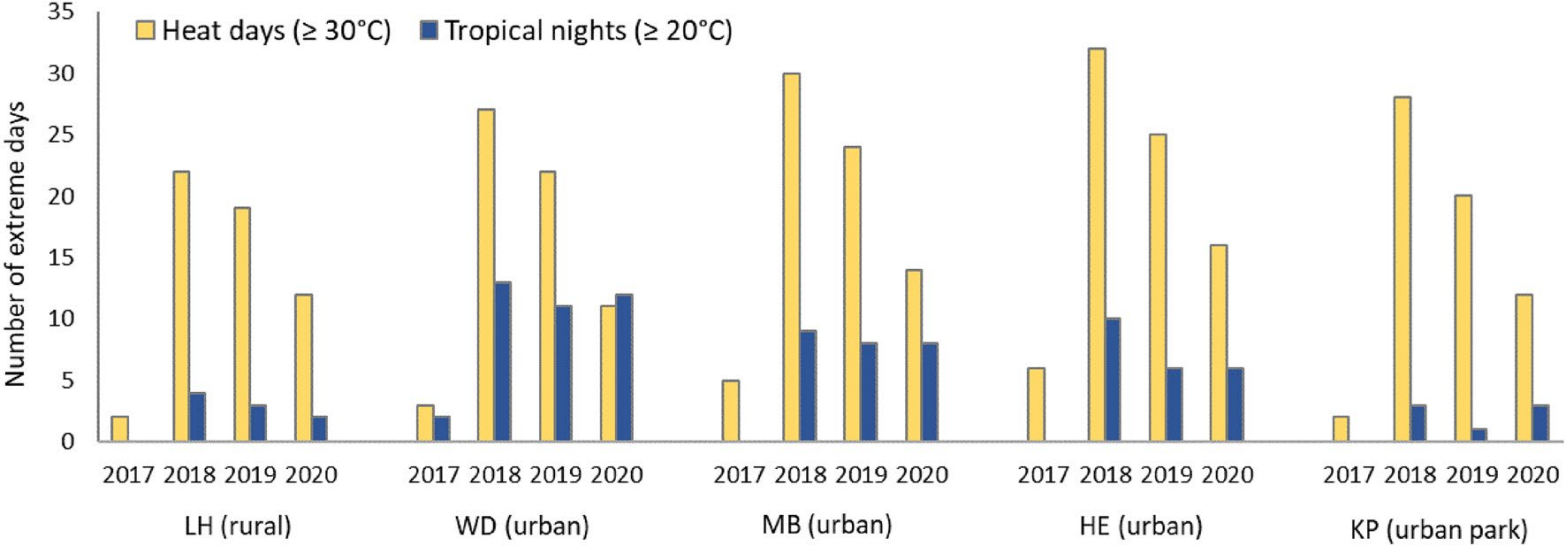
Total number of heat days (in yellow) and tropical nights (in blue) measured at the rural, urban and urban park measurement sites for the summer periods of 2017, 2018, 2019, 2020. Note: LH = Langenhagen, WD = Weidendamm, MB = Marianne-Baecker Allee, HE = Herrenhausen, KP = Kattenbooks Park.

Figure 2 shows the total number of heat days and tropical nights for all measurement stations for the years 2017, 2018, 2019 and 2020. The number of heat days was lowest for the non-heat year 2017 (between 2 heat days at LH and 6 at HE) and highest for 2018 (between 22 heat days at LH and 32 at HE) for all measurement stations. Numbers are higher respectively for the inner-city urban sites compared to the rural site LH. The total numbers differ, however, with regard to the tropical nights. For 2017, the inner-city urban site of WD is the only measurement station where tropical nights were detected (2 in 2017). During the heat years of 2018–2020, the total number of tropical nights was two up to four times higher for the inner-city urban stations compared to the rural station LH and also compared to the urban park station, indicating an UHI effect during night-time summer conditions for the years of unprecedented heat. The highest number of topical nights was shown for the year 2018 at all measurement stations (with 3 tropical nights at the urban park station KP, 4 at the rural station LH and 13 at the urban station WD).

**Figure 3:**
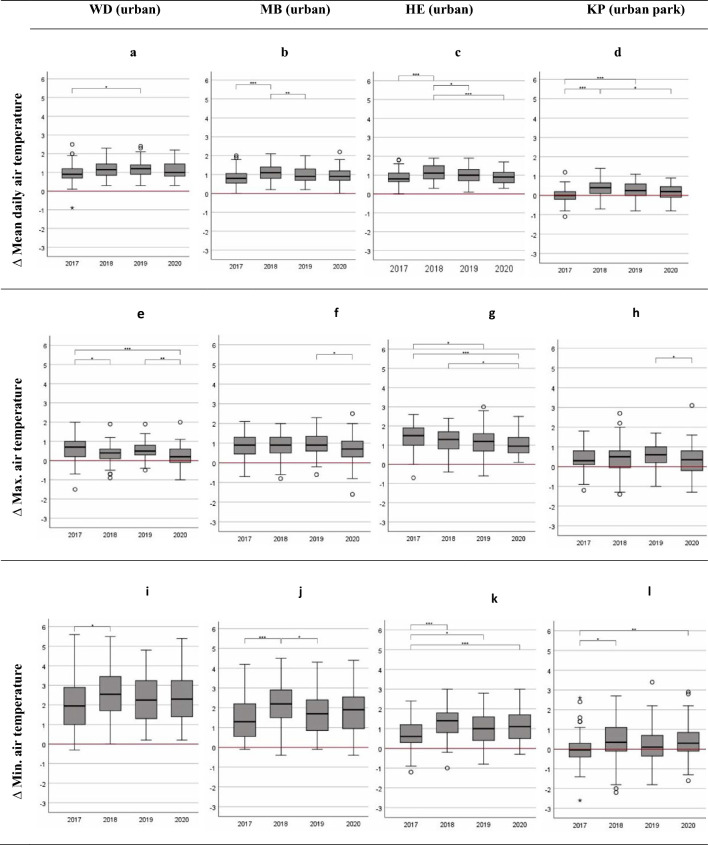
Boxplots of the mean differences of daily mean air temperature, maximum air temperature and minimum air temperature between the rural and the urban sites for each year of interest. Whiskers indicate 1.5 × interquartile range. Significant differences are indicated between the years with * at the 0.05 level, ** at the 0.01 level, and *** at the 0.001 level. Note: In cases when Levene statistics revealed that the variances for the variables were not equal, Welch statistic was reported and Tamhane’s T2 – post-hoc test were used as a robust multiple comparison tests, not assuming equal variances. Note: LH = Langenhagen, WD = Weidendamm, MB = Marianne-Baecker Allee, HE = Herrenhausen, KP = Kattenbooks Park.

To assess whether the UHI intensity is significantly different between all years of interest, Fig. 3 shows boxplots of daily mean air temperature, maximum and minimum air temperatures and also highlights indication of significance by different significance levels.

Significant differences for the daily mean air temperature were observed for the inner-city urban sites for the non-heat year of 2017 but on a lower level compared to 2018 (Fig. 3, boxplots a–d). Significant differences were found between 2017 and the heat years when minimum air temperatures indicating night-time conditions are considered. The greatest differences were identified for 2018 compared to 2017 for the urban sites WD, MB and HE, reaching up to around 2.5 K (median value, Fig. 3, boxplots i–l), indicating that the UHI intensity is pronounced during the night in years of extreme heat when compared to a non-heat year. This association of a synergistic effect of the UHI and heat wave periods is, however, not detectible and even inverted when considering the differences in the daily maximum temperatures (Fig. 3, boxplots e–h). In 2017, the UHI intensity is even on higher levels for maximum air temperatures compared to the heat years of 2018, 2019, and 2020, with significant differences particularly for the urban sites of WD and HE. The difference between the years is non-significant when the urban park site is considered.

### Land use and land cover (LULC) structures around the measurement sites

**Figure 4:**
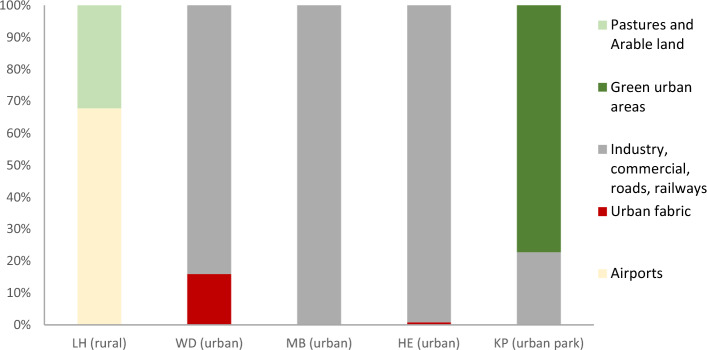
Percentage share of LULC in a 100 m buffer around the measurement sites. Note: LULC classes are summarized based on Urban Atlas 2018 classification. Urban fabric includes “Continuous urban fabric” and “Discontinuous medium density urban fabric”. Note: LH = Langenhagen, WD = Weidendamm, MB = Marianne-Baecker Allee, HE = Herrenhausen, KP = Kattenbooks Park.

The percentage shares of LULC within a buffer of 100 m around the measurement sites are shown in Fig. 4. LULC around the rural measurement station LH consists of open areas such as pastures, arable land and land denoted as airport. The inner-city urban measurement sites are mostly surrounded by industrial, commercial and transport infrastructure areas. The share of urban fabric is around 16% at the urban station WD and 1% at the urban station HE. More than three quarters of the LULC close to the urban park station (KP) is classified as green urban areas and around 20% as transport infrastructure.

**Figure 5:**
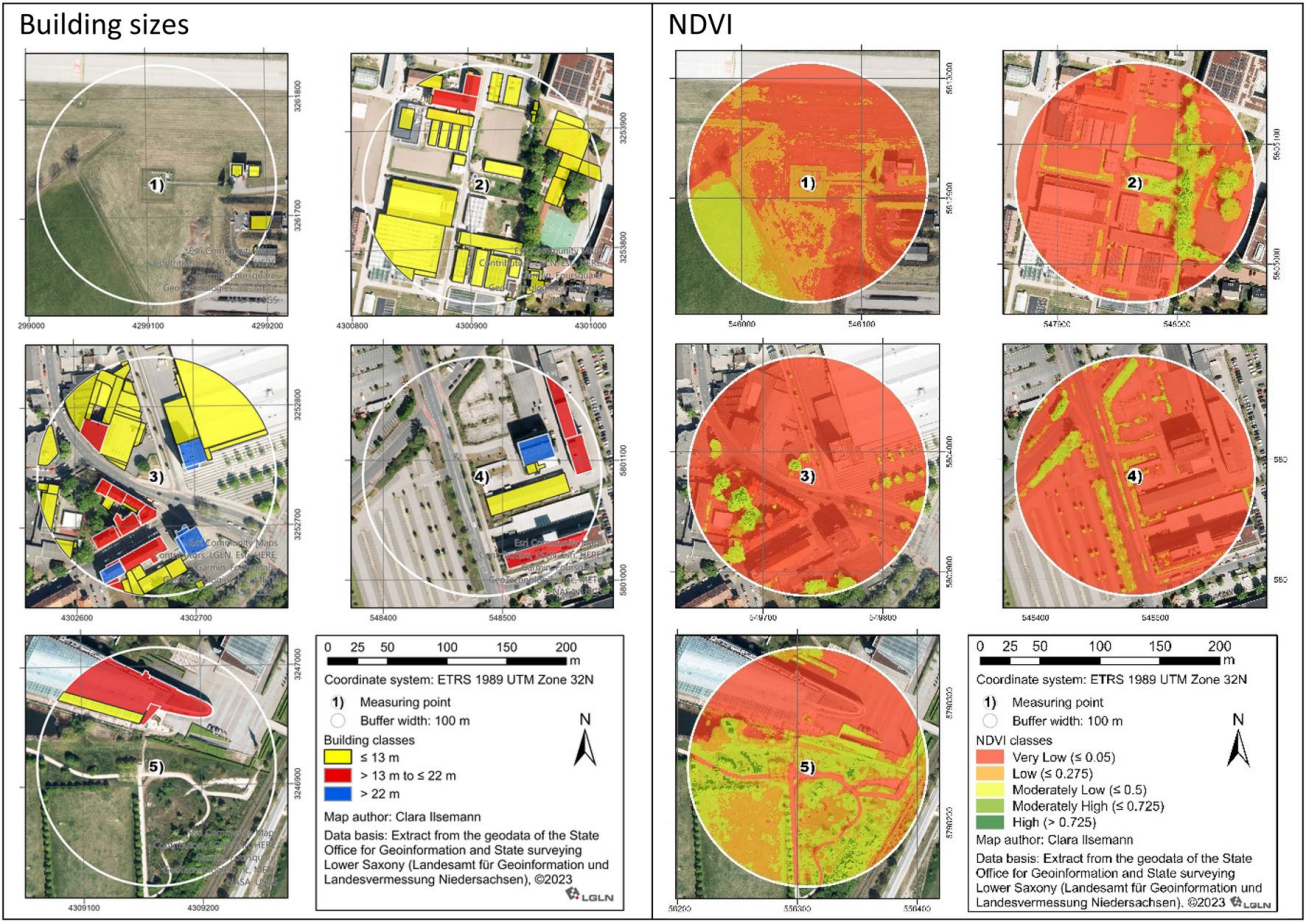
Buildings with different heights within a 100 m buffer around the measurement stations (left) and NDVI values (right). Note: (1) LH = Langenhagen (rural site); (2) HE = Herrenhausen (urban site), (3) WD = Weidendamm (urban site); (4) MB = Marianne-Baecker Allee (urban site); (5) KP = Kattenbrookspark (urban park site).

The respective LULC is also reflected in the NDVI values illustrated in Fig. 5. Percentage of areas with higher NDVI values indicating more vegetation cover are shown for the rural site (LH) and for the urban park site (KP). The area around the inner-city urban measurement stations is mostly sealed, as shown by a high share of the area having NDVI values close to or below 0.05. The inner-city urban stations HE and WD also show a high share of building area with more than 50% of the area covered by smaller buildings below 13 m height.

### Synthesis of results

Table 4 presents a synthesis of the main variables with regards to the extent of the UHI intensity in our study. It shows the data for 2017 compared with 2018 as a heat year. The rural station LH and the urban park station KP are presented with the lowest values for the maximum and minimum air temperatures for both years. The highest measured air temperature values are indicated for the inner-city urban stations, with HE showing the highest maximum daily values, and WD showing the highest minimum air temperatures for both years. The number of heat days and tropical nights were lowest for 2017 and 2018 for the rural and the urban park stations. The number of heat days was highest with the urban station HE (32 in 2018), while the number is slightly lower for the urban station WD (27 in 2018). At this measurement station (WD), the number of the tropical nights is, however, highest (13 in 2018 vs. 10 at HE). Also, at this measurement station (WD), the relative humidity was lowest in both years. This difference between the two urban stations WD and HE may be explained by the different LULC structures at WD compared to HE. At WD, the area within 100 m shows the highest share of impervious surface (indicated by very low NDVI values < 0.05, Fig. 5) and the highest share of buildings, in particular buildings taller than 13 m (Table 4).

**Table 4:** Overview of main variables related to UHI intensity for 2017 and 2018. Note: LH = Langenhagen, WD = Weidendamm, MB = Marianne-Baecker Allee, HE = Herrenhausen, KP = Kattenbooks Park.

		LH (rural)	WD (urban)	MB (urban)	HE (urban)	KP (urban park)
Air. Temp max./min. (°C)	2017	**33.1**/**5.7**	33.9/***9.9***	34.6/8.9	***35.2***/7. 3	33.3/5. 9
	2018	**36.5**/6.7	37.1/***9.7***	37.8/8.5	***38.0***/8.6	36.7/**6.5**
Heat days (No.)	2017	**2**	3	5	***6***	**2**
	2018	**22**	27	30	***32***	28
Tropical nights (No.)	2017	**0**	***2***	**0**	**0**	**0**
	2018	4	***13***	9	10	**3**
Rel. humidity (%)	2017	**75.4** (9.2)	***67.5*** (10.7)	67.8 (10.4)	72.7 (9.1)	73.2 (9.0)
	2018	65.4 (11.5)	***57.4*** (12.9)	59.9 (12.6)	62.5 (11.1)	**62.8** (12.2)
NDVI (% of area)	≤ 0.275	89.19	95.10	*95.47*	90.50	**65.68**
	> 0.275	**10.81**	***4.90***	**4.53**	9.50	*34.32*
Buildings (%)	< 13 m	**1.17**	29.13	3.88	***32.22***	1.93
	≥ 13 m	**0.00**	***11.00***	7.54	2.32	10.20
	Total	**1.17**	***40.13***	11.42	34.65	12.13

Values in bold show lowest values and in bold italics highest values. This is inversed for relative humidity.

Based on these LULC conditions, we conclude that the area around the urban station HE is heating up to higher levels during the day compared to WD, but also cools down more during the night. At the urban station WD, the daily air temperature is heating up to a slightly lower degree, but also cools down less during the night due to a much slower heat release. The slower heat release can be explained by the high share of impervious areas and building cover. In particular, the comparatively higher share of buildings with heights of 13 m and above and the high share of impervious surfaces prevent nocturnal cooling. The tall buildings provide, however, more shading throughout the day which can explain that the maximum heat and number of heat days is comparatively lower here at WD compared to the other inner-city urban stations.

## Discussion

In this study, we analysed the extent of the UHI effect for the city of Hannover by comparing three years of unprecedented summer heat (2018–2020) with a non-heat year (2017). Using data from five measurement stations located in a rura areal, three inner-city urban areas and an urban park area, we identified the UHI phenomenon for all years under study and for all measurement stations. When comparing air temperatures of the non-heat year with the extreme heat years, we found a greater UHI intensity during the nights of the extreme heat years compared to the non-heat year when minimum air temperatures are considered. This association was detectible especially for the very hot and very dry summer of 2018. Conversely, when day-time temperatures are considered with maximum air temperatures, the UHI intensity was on a lower level during the heat years compared to the non-heat year. The identified UHI intensity associations were on a lower and partly non-significant level for the urban park location indicating the local cooling potential of urban green spaces to mitigate the UHI.

The years of 2018, 2019 and 2020 have been highlighted as years with exceptional, unprecedented heat in Europe and Germany^6,38^. In particular, 2018 was indicated as the warmest and also driest year in Germany since weather records. We identified the greatest amplification of the UHI intensity under heat conditions for night-time conditions for 2018. The extreme conditions particularly in 2018 may be related to the co-occurrence of the heatwave and drought period which could have led to further synergistic effects amplifying the heat conditions. Elsewhere, in Europe^38^ and China^2^, the co-occurrence of heat and drought have been shown to amplify the effects of both on biodiversity and vegetation conditions, such as by limiting the provision of ecosystem services.

For Hannover, we identified an air temperature amplification effect of the UHI for the night-time conditions for the heat years. For day-time conditions (maximum temperatures), we saw a reduced intensity of the UHI compared to the non-heat year. For the city of Berlin, Germany, during the heatwaves of 1996 and 2006^24^, an amplified UHI was also identified for night-time conditions with an intensity of 1.29 K for 1994 and 0.83 K for 2006. When day-time temperatures were considered, air temperature measured at the Berlin city locations were even lower than those in the rural areas. Another study in Berlin found a non-significant day-time UHI intensity between normal conditions and heat days^39^. In the city of Guangzhou, China, the UHI intensity under heat waves was found to be amplified during the day and also the night but that it was also more significant and stronger at night^23^. Others have found that the UHI is stronger during heat days for both day-time and night-time conditions, such as in an Australian study for Adelaide and Melbourne^10^.

The different UHI intensities for day-time and night-time conditions may be related to the characteristics of the local urban environment including the LULC close to the measurement stations. Urban structures with urban materials, high density of buildings, and high rates of impervious surfaces contribute to a high heat storage capacity during the day. The thermal admittance and release of heat in the form of sensible heat is, then, also higher during the night. Together with a usually more stable nocturnal boundary layer and a decreased vertical mixing, the UHI intensity shows highest values at night. This effect is reinforced during extreme heat periods^23^ which we also found for Hannover, particularly in 2018. Similarly to our results of the inner-city urban location of WD having the highest share of taller buildings, research for the city of Guanzhou found that local climate zones with higher shares of taller and compact buildings had higher air temperatures but on a slightly lower level when compared to other urban climate zones^23^. The differences were explained by possible shading effects of taller buildings.

For Hannover, we showed reduced air temperatures with lowest minimum values during the day and the night for the rural measurement station but also for the urban park station, indicating the importance of urban green spaces to mitigate local UHI effects and the resulting impacts on human health. The UHI effect was nearly completely diminished for the urban park site. The effect of vegetation, such as green spaces, on local air temperatures and increased relative air humidity was also found in a study of the city of Würzburg, Germany^11^^.^ In this study, Rahman et al. highlighted, however, that the cooling effect of a green space in urban areas is dependent on the local built environment and urban morphology, which may reduce wind speed, hinder ventilation and vertical mixing counteracting a potential cooling effect. For Hannover, the urban park measurement site is located in an open, lawn covered park area with a very low number of buildings surrounding it. The usual blocking of the natural wind flow in dense inner-city areas is reduced here but allows heat release during the night. The urban park site in Hannover can, thus, be regarded as a local cooling island under heat particularly during the night. This cooling effect is local, however, and has a limited effect on the wider residential area^38^ and has also been referred to as the “tree cooling pond effect”^40^. Referring to the median radiant temperatures, Zhang et al. (2023) showed that the cooling effect of individual trees was mostly identified for the crown-canopied spaces with very limited effects when distance from the trunk increased in more sunny places^40^.

For a more climate resilient urban planning, our results highlight the need to introduce structurally diverse urban green spaces over an entire city area at as many locations as possible. Trees provide a cooling function particularly during the day even under hot and dry conditions due to shading effects^38^. Open areas such as lawn and grassland areas promote heat release and, thus, cooling during the night^38^. The combination of both, tree covered and open areas, for structurally diverse urban green spaces has been introduced as the “savannah approach”^38,41^. When developing and maintaining urban green spaces, also the wise selection of climate (heat and drought) adapted tree species with larger canopies could help to mitigate the UHI^40^. As space in growing cities is contested, novel concepts such as pocket parks, smaller parks in street corners, brownfield redevelopments or additional green infrastructure elements such as green roofs and facades could complement a wide and divers urban green space network^38,42^.

### Limitations

Our analyses focussed on summer periods and used data of fixed reference dates (1 June and 31 August). Temperature extremes, however, potentially occur earlier (in May) or later (September) in the year. Due to this temporal focus, we may have missed out some additional hot days or tropical nights. The measurement stations also had some missing or invalid data, although with the overall data loss was below 1%.

Our study is based on data from five fixed measurement stations. These locations have different site conditions, so that a comprehensive picture of the complexity of the entire city cannot be provided. The data includes, however, several inner-city urban sites and an urban park site and the respective environmental conditions in terms of LULC were described and temperature differences explained in detail. Other approaches apply the concept of the footprint effect of an UHI using remote sensing data and land surface temperature which enables the application of different buffer zones to detect temperature differences^43^. Still the usability of remote sensing data is depending on cloud cover, etc. Finally, we used the NDVI as an indicator for LULC, in particular to show unsealed, vegetated areas. The values of the NDIV are, however, depending on the fitness of the vegetation and lower values can occur when vegetation is impacted e.g. during drought periods.

## Conclusions

In this study, we analysed the extent and intensity of the UHI in the city of Hannover during unprecedented heat conditions. We compared data for the extreme summer heat periods of 2018–2020 with data from the non-heat year 2017. We identified an UHI for all years under study but a greater UHI intensity within the heat years compared to the non-heat year when night-time temperatures are considered. Conversely, we found a lower UHI intensity when focussing on the daily temperatures during the heat vs. non-heat years. These UHI intensity associations are strongest for the inner-city urban measurement sites located in dense urban districts. The measurement site at the urban park location showed a lower UHI and partly non-significant difference among the different years.

The results indicate the importance of implementing and maintaining urban green spaces in cities as local cooling zones which will become even more important under ongoing global warming. With increasing temperatures and higher frequencies of extreme heat events, the provision of structurally diverse urban green spaces with tree and tree-canopy covered areas to provide shading during the day but also open spaces to allow cooling during the night can support heat mitigation measures to regulate the local urban climate.

## Data Availability

Data will be made available on request but based on permission of the Deutsche Wetterdienst (see Acknowledgements section).
